# Being asked to tell an unpleasant truth about another person activates anterior insula and medial prefrontal cortex

**DOI:** 10.3389/fnhum.2015.00553

**Published:** 2015-10-20

**Authors:** Melissa M. Littlefield, Martin J. Dietz, Des Fitzgerald, Kasper J. Knudsen, James Tonks

**Affiliations:** ^1^Department of Kinesiology and Community Health, Department of English, and The Beckman Institute, University of Illinois at Urbana-ChampaignUrbana, IL, USA; ^2^Center for Functionally Integrative Neuroscience, Institute of Clinical Medicine, Aarhus UniversityAarhus, Denmark; ^3^School of Social Sciences, Cardiff UniversityCardiff, UK; ^4^Hubbub—The Hub at Wellcome CollectionLondon, UK; ^5^Section for Anthropology and Ethnography, Department of Culture and Society, Aarhus UniversityAarhus, Denmark; ^6^Department of Psychology, University of LincolnLincoln, UK; ^7^Dame Hannah Rogers TrustExeter, UK

**Keywords:** fMRI, social reasoning, theory of mind, truth telling, social evaluation

## Abstract

“Truth” has been used as a baseline condition in several functional magnetic resonance imaging (fMRI) studies of deception. However, like deception, telling the truth is an inherently social construct, which requires consideration of another person's mental state, a phenomenon known as Theory of Mind. Using a novel ecological paradigm, we examined blood oxygenation level dependent (BOLD) responses during social and simple truth telling. Participants (*n* = 27) were randomly divided into two competing teams. Post-competition, each participant was scanned while evaluating performances from in-group and out-group members. Participants were asked to be honest and were told that their evaluations would be made public. We found increased BOLD responses in the medial prefrontal cortex, bilateral anterior insula and precuneus when participants were asked to tell social truths compared to simple truths about another person. At the behavioral level, participants were slower at responding to social compared to simple questions about another person. These findings suggest that telling the truth is a nuanced cognitive operation that is dependent on the degree of mentalizing. Importantly, we show that the cortical regions engaged by truth telling show a distinct pattern when the task requires social reasoning.

## Introduction

Telling the truth is not a simple cognitive task. Honesty requires assessment of individual social situations and, in particular, consideration of another person's mental state, a phenomenon known as Theory of Mind (Frith, 2007). Honesty about another person, in particular (and especially being brutally honest about that person), sometimes also requires us to put ourselves in that person's shoes, and to emotionally regulate ourselves accordingly—to empathize, in other words (Decety and Jackson, 2004; Krämer et al., 2010). Thus, certain social situations make honesty particularly challenging: confessions of wrongdoing (Hilgendorf and Irving, 1981; Deslauriers-Varin et al., 2011), difficult medical diagnoses (Buckman, 1984; Fallowfield and Jenkins, 2004; Shaw et al., 2013), and statements that contradict dominant ideological or political points of view (Brounéus, 2010). Anecdotally, these situations can cause us to blush, experience increased respiration, or feel our hearts beating more rapidly. The scale and scope of truth telling can be as simple as responding “yes” or “no” to a simple question, or as complex as admitting guilt in a criminal-legal situation. Furthermore, personal and cultural norms play a role in definitions of truth: valances attached to being honest are very different for different people and in different social situations (Bok, 1999; Zahedi, 2011). Thus, not only the definition of what a truth is, but also the experience of being asked to tell the truth, as well as the cognitive load *of* that telling, are very often mediated by social contexts: in short, humans assess complex social situations when faced with telling the truth (Sip et al., 2013).

However, despite this social contingency, cognitive neuroscience studies often consider “truth” to be a clearly defined control condition. For example, some notable prior studies on neurobiological and cognitive components of deception attempted to control for social context(s), and in so doing, have taken truth conditions to be neutral baselines that can used to control for deceptive responses. Studies with more nuanced designs assumed truth as a baseline, in part, because it was not a variable of interest (Langleben et al., 2005; Bhatt et al., 2009; Lee et al., 2010); other studies disaggregated truth conditions from deception conditions on the basis that truth is a less active condition than deception (Spence et al., 2001, 2004; Langleben et al., 2002; Kozel et al., 2004; Debey et al., 2012; Ito et al., 2012; Vartanian et al., 2012). With few exceptions (Mohamed et al., 2006; Marques et al., 2009; Verschuere et al., 2010; Hadar et al., 2012), truth telling has not been studied as a socially complex variable in recent fMRI studies. Indeed, one recent study characterizes its “truth/truth telling state” as “inactive” (Hu et al., 2012: 6). While researchers have begun to treat deception within “a framework of social decision-making (see e.g., Abe et al., 2007; Barrios et al., 2008; Baumgartner et al., 2009; Greene and Paxton, 2009; Bhatt et al., 2010; Carrión et al., 2010; Sip et al., 2010)” (Sip et al., 2012), the same cannot yet be said of truth telling. Yet, truth telling appears to meet Sip et al.'s (2012) criteria for a “complex social interaction”—in other words, it “involves a broad set of cognitive processes, including the ability (i) to determine the possible courses of action and to identify how they could be coordinated with the interlocutor, (ii) to weigh these available courses of action against one another, and (iii) to choose which action to perform next in the interaction” (Sip et al., 2012).

Our study began with two hypotheses: (1) we hypothesized that being asked to tell the truth engages areas of the brain that have previously been associated with mentalizing, specifically the medial prefrontal cortex (Amodio and Frith, 2006) and the insular cortex (Bernhardt and Singer, 2012), but only when truth telling required social reasoning compared to factual reasoning. This was based on the hypothesis that telling the truth in a social context requires predicting the mental point of view of another person and empathizing in terms of predicting the feelings of that person; (2) we hypothesized that these processes would be pronounced when telling the truth about in-group compared to out-group members. Hypothesis (1) was confirmed; hypothesis (2) was not confirmed.

To test these hypotheses, we examined BOLD responses during truth telling that required complex social, inter-personal calculation, compared to truth telling about simple and impersonal physical attributes. The study design consisted of an ecological experiment that allowed us to create a realistic, yet controlled social situation among the participants. The experiment took place over 3 days. During this period, participants established team identities by competing against one another as members of two distinct choir-teams. After the competition, participants evaluated each other's singing performances in the MRI scanner. Crucially, participants believed that their evaluations would become public at a reunion luncheon, and were asked to “be honest” in their evaluations of fellow participants who had performed poorly. However, the only people who were actually evaluated were four actors, two men, and two women on each team. Participants were not informed that there were actors among them, and the actors were instructed to play the role of a participant without revealing their identities as actors. Further, the actors were instructed to be very likable, but to sing very poorly. Participants reported strong team-identification throughout the first day of the experiment. During a post-scan debriefing, nearly all participants were conscious of their answers and their revelation at the reunion luncheon. All participants believed that they were evaluating fellow participants.

## Materials and methods

### Participants

Twenty-seven healthy volunteers (21 females) aged 20–35 were recruited from local choirs in and around Aarhus, Denmark. We chose to recruit choir singers as participants for the study for two reasons: first, choir singing involves people's ability to perform individually within a collective, that is, individual singers must adjust their own voice to fit a group performance while simultaneously assessing the performance of their fellow singers. Second, as the study took place in Aarhus, Denmark, which is home to many different choirs, we found that choir singing adapted well to the social and cultural setting of the experiment. All participants completed “Day 1” of the study involving team-building. Three participants were excluded due to metal implants and one for anxiety. The remaining 23 volunteers (5 male, 18 female) participated in “Day 2” and fMRI scanning. Participants provided written informed consent as approved by the University of Illinois, Urbana-Champaign Institutional Review Board (#10084) and the Ethics Committee of the Central Denmark Region.

### Procedure

The experiment included two parts that were inseparable: Day 1 consisted of an 8-h team-building behavioral experiment. Day 2 consisted of a 1-h fMRI session. Participants were led to believe that there would also be a Day 3 at a future date, in which all participants would be reunited with one another, their evaluations of each others performances would be revealed, and a winning team would be chosen.

Day 1:

- Participants arrived together at the Department of Music at Aarhus University, Denmark, at 10:00 am. They gave written informed consent and were administered a Hospital Anxiety and Depression Measure (HADS) (Zigmond and Snaith, 1983). All participants passed the HADS and were approved by the on-team clinical psychologist (Tonks) for the experiment. The HADS measure ensured that our sample population was normal, healthy, and able to participate in a paradigm that was designed to create experimental stress.
- Participants were then broken into two teams with equal proportions of males and females on each team. The experimenters chose teams in a random-seeming manner, but allocated one female and one male actor to each team. Participants remained unaware throughout that the actors were not normal participants.
- Participants each received a colored bandana (blue or purple) to identify their team.
- Each team retired to a separate room for a team-building exercise (i.e., the “Tree of Life”), which allowed participants to share personal narratives about their backgrounds, present circumstances, and future goals, before returning to a common room for lunch, where the teams sat separately.
- Both teams competed in a team-based quiz game concerning various music-related questions. Participants were told that the results would be counted toward the team competition, which would be announced during the “Day 3” reunion.
- Each team then met their respective choir coaches and, in separate rooms, practiced one song to be sung competitively.
- Participants (including the actors) were individually removed from the group to film a 1-min singing video. Although no video footage of the participants was actually recorded (aside from that of the actors, who were aware of the deception), all participants were informed that other participants from both teams would view these videos before the fMRI scan on Day 2, and that a randomly-chosen video would be the basis of each participant's fMRI evaluation task.
- Teams came together for the final competition, which was judged by a well-known choir director from Aarhus; results were not provided, but participants were told that the scores would count toward the larger, team competition.
- Teams went to dinner at separate restaurants to further bond.

Day 2:

- Participants arrived individually for the fMRI experiment at the Aarhus University Hospital and gave their informed written consent.
- Participants were led to believe that they were drawing names at random from a hat (in fact, the hat was rigged).
- Participants all viewed the same four videos of the likable, yet poorly-singing actors (two from each team, one female and one male).
- Participants were asked to be as honest as possible in their evaluations of the singers, but also reminded that their evaluations would be made public at the reunion lunch on “Day 3.”

### Experimental paradigm

**Figure 1:**
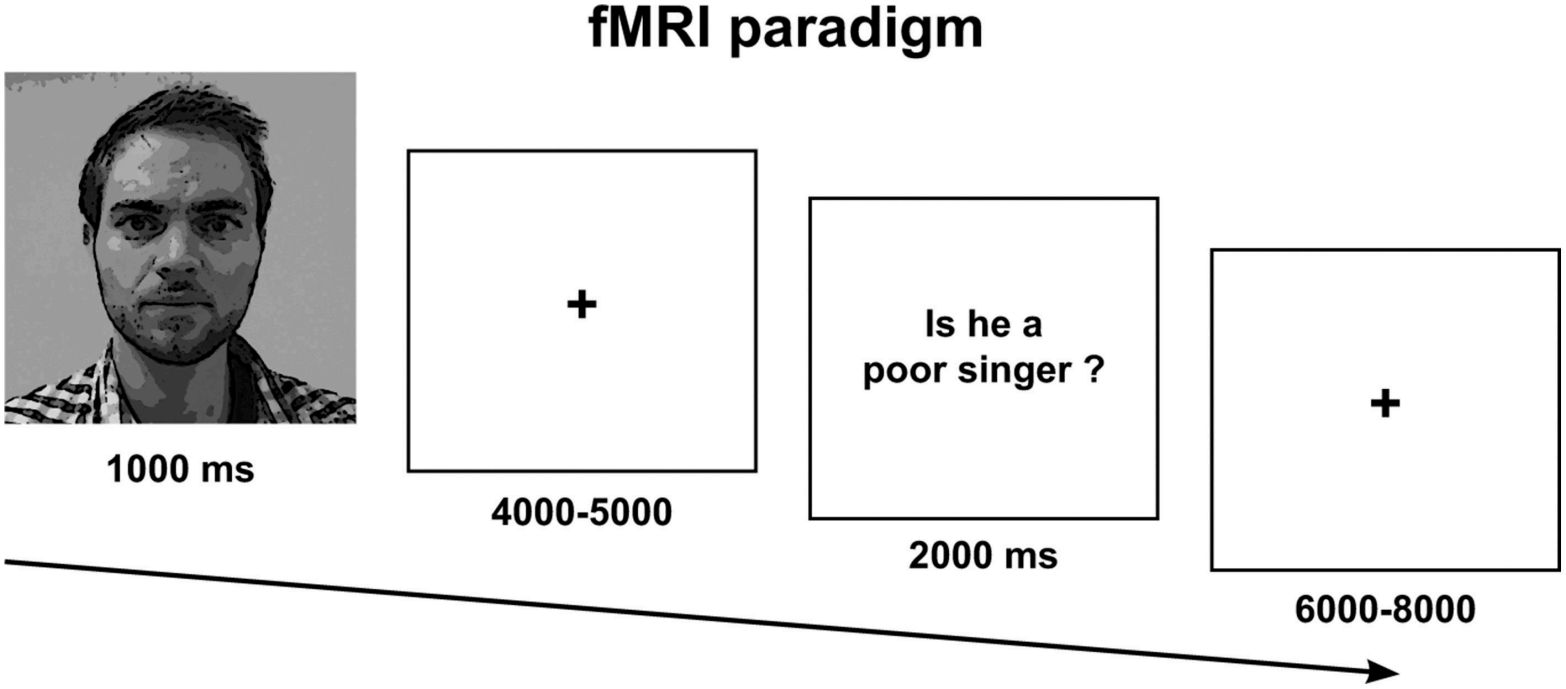
**Experimental paradigm**.

The fMRI paradigm comprised a 2 × 2 factorial design with the following conditions: Social Truth-Ingroup, Social Truth-Outgroup, Simple Truth-Ingroup, Simple Truth-Outgroup. Each condition comprised an epoch that started with a picture of the fellow participant followed by a question about her or him. A fixation cross was displayed between faces and questions with variable duration to induce a jittered inter-stimulus interval and increase design efficiency (Figure 1). A *social* question was one that requested a subjective and evaluative opinion about the participant, e.g., “is Person X a poor singer?” A *simple* question did not require reasoning about the person beyond his or her physical appearance and did not put the participant's social relationships at stake for the formulation of a truthful response, e.g., “does Person X have brown hair?” The social and simple questions were formulated and validated in a separate pilot study in which 18 college-aged participants rated 40 possible questions on a social stressfulness scale from 1–5. Each scan lasted 20 min and comprised a total of 80 randomized epochs. The subjects were required to answer “yes” with their right-hand index finger or “no” with their middle finger. Note that the answers were deliberately subjective and hence we took no measure of the accuracy of evaluations (a limitation that we discuss further below). The prediction that participants would give genuine or authentic responses responses was highly dependent on the ecological validity of the experimental situation that we had created. After the fMRI session, we revealed all deceptive elements of the experiment to the participants during a de-brief.

### fMRI acquisition

Functional images were acquired on a 3 Tesla Siemens Trio MRI Scanner (Erlangen, Germany) using a 32 channel RF head-coil. T2^*^-weighted echo planar images (EPI) consisting of 43 slices of 3 mm thickness per volume were acquired in interleaved fashion with the following parameters: repetition time (TR) = 2.14 s, echo time (TE) = 27 ms, flip angle = 90°, field of view (FOV) = 192 × 192 mm, and in-plane resolution = 64 × 64. Soft cushions were used to minimize head movements. In parallel with EPI time-series, the participants' pulse and respiration were recorded using infrared pulse oximetry on the index finger and a pneumatic thoracic belt (Siemens, Erlangen, Germany).

### fMRI data analysis

#### Single-subject analysis

fMRI data analysis was performed using Statistical Parametric Mapping (SPM8, revision 4667). The functional images of each subject were realigned (Friston et al., 1995a), spatially normalized to MNI space using the EPI template (Ashburner and Friston, 1999), and smoothed with a Gaussian kernel of 8 mm FWHM. The time-series in each voxel was high-pass filtered at 128 s using a discrete cosine set to remove low-frequency drifts. Statistical analysis was implemented using a general linear model (Friston et al., 1995b). Regressors encoding the experimental design were convolved with a canonical hemodynamic response function and fitted to the fMRI time-series. This also included a partition of no interest explaining the mere effects of visual stimuli and motor responses related to the button press. Serial correlations due to physiological noise were modeled using Nuisance Variable Regression (Lund et al., 2006), an alternative to the standard first-order autoregressive AR(1) model. Physiological oscillations that affect the hemodynamics, such as pulse (1 Hz) and respiration (0.2 Hz), are usually present in the EPI time-series in the form of aliased higher-frequency components expressed at lower frequencies due to undersampling at typical TRs of 0.5 Hz. The design matrix thus included a partition of nuisance regressors that reflected pulse and respiration in the form of their (theoretical) aliases and a partition modeling the instantaneous effect of head movement, its spin excitation history and their 2nd-order expansions (Friston et al., 1996; Lund et al., 2006).

#### Group analysis

Linear contrast images were created for each subject testing for the main effects and the interaction of the 2 × 2 experimental conditions during the decision period when subjects responded to the question. In order to make inferences at the population-level, we performed random-effects analyses for the two main effects and the interaction using one-sample *t*-tests. All statistical tests were thresholded at *p* < 0.05, family-wise error (FWE) whole-brain corrected for multiple comparisons using Random Field Theory (Worsley et al., 1996).

## Results

### Behavioral responses

**Figure 2:**
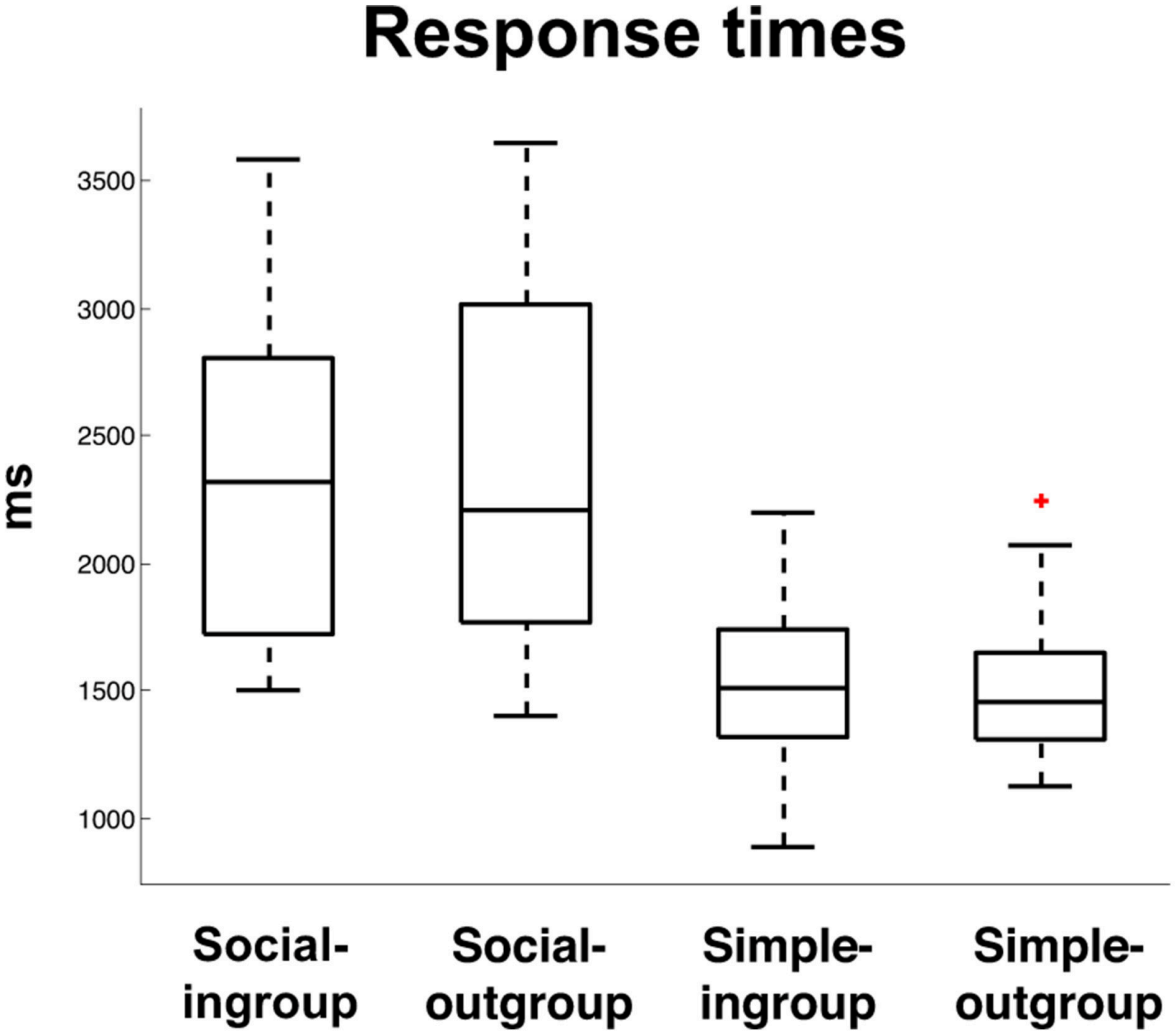
**Response times (ms) to Social Truth-Ingroup, Social Truth-Outgroup, Simple Truth-Ingroup, Simple Truth-Outgroup**. The “+” indicates a single outlier with respect to the upper quartile of the data.

When formulating a response to a social question, subjects had significantly slower response times compared to simple questions, *t*_(66)_ = 13.5, *p* < 0.0001. There was no effect of In-group > Out-group on reaction times, *F*_(1, 66)_ = 0.04, *p* = 0.83, nor was there an effect of the interaction, *F*_(1, 66)_ = 0.08. *p* = 0.77. This behavioral finding supports the hypothesis that social truth telling, like deception, is a more complex task than simple truth telling, which places higher demands on executive functions (Figure 2).

### fMRI results

**Figure 3:**
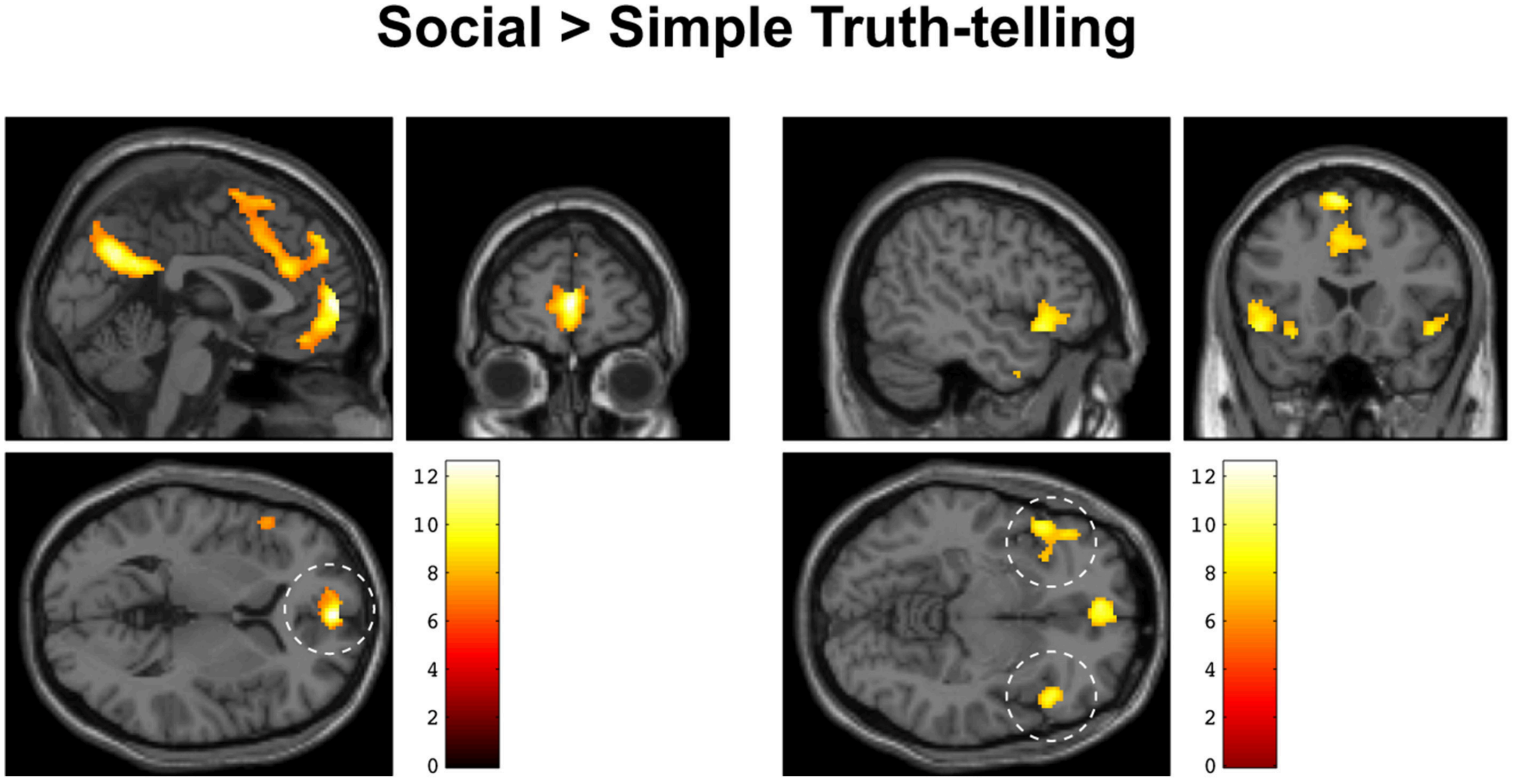
**Increased BOLD activity during social > simple truth telling overlaid on an anatomical MRI of a single subject in MNI space**. **Left**: sagittal, coronal, and axial sections centered on the medial prefrontal activation (dashed circle). **Right**: sagittal, coronal, and axial sections centered on the activation in bilateral anterior insula and inferior frontal gyrus (dashed circles).

The fMRI analysis focused on the short decision period following a question to the point when subjects responded to either a social or a simple truth question about an in-group fellow or an out-group fellow (see Figure 1). In this way, we were able to isolate the period during which participants decided how to respond, controlling for the mere effects of finger movement. We found a significant effect of social > simple truth telling in medial prefrontal cortex, bilateral anterior insula, inferior frontal cortex, and the precuneus. Clusters with >100 contiguous voxels were located in the ventral part of the medial prefrontal cortex with peak at MNI: [0 58 4], *t*_(22)_ = 12.55, *p* < 0.001, left anterior insula with peak at MNI [−48 20 −8], *t*_(22)_ = 9.78, *p* < 0.001, right anterior insula with peak at MNI [48 24 −8], *t*_(22)_ = 9.34, *p* < 0.001, left supplementary motor area with peak at MNI [−6 18 64], *t*_(22)_ = 10.71, *p* < 0.001 and the precuneus with peak at MNI [2 −66 32], *t*_(22)_ = 11.69, *p* < 0.001 (Figure 3). There was no significant main effect of Ingroup > Outgroup, no effect of simple truth, nor was there an effect of the interaction.

## Discussion

The goal of this study was to use fMRI measures to investigate the social-cognitive nuances of truth telling. This entailed testing whether the cortical responses that mediate truth telling are sensitive to social context and whether these responses are affected by group identity. We hypothesized that social truth telling, compared to simple truth telling, would engage brain regions previously associated with social cognition. Specifically, we anticipated activity in the medial prefrontal cortex (Amodio and Frith, 2006) and the insula (Bernhardt and Singer, 2012). This was based on the intuition that telling the truth, specifically in a social context, requires predicting the mental point of view of another person and empathizing in terms of predicting the feelings of that person. We further hypothesized that these processes would be pronounced when telling the truth about in-group compared to out-group members.

In support of our first hypothesis, our findings show that formulating a response to social questions increases activity in the ventral medial prefrontal cortex, anterior insula, and precuneus, compared to formulating a response to simple factual questions. Activation of these regions is accompanied by significantly slower reaction times to social questions, both during in-group and out-group conditions. This indicates that being asked to tell the truth under conditions of potentially adverse social outcomes, may be associated with response inhibition, mediated by the anterior insula and prefrontal network. This activation pattern is almost identical to the pattern reported in well-controlled studies of deception. Such activation has also been associated with increased activation of areas that have been associated with mentalizing (Amodio and Frith, 2006), third-person perspective-taking (Vogeley et al., 2004), and empathy (Bernhardt and Singer, 2012).

We did not find any support for the second hypothesis concerning the in-group/out-group distinction in either the behavioral data or the fMRI data. We used an in-group/out-group paradigm because we hypothesized that this difference in social context would modulate the effects of social truth telling. While the hypothesized difference between in-group and out-group was not expressed in reaction times, the neuronal processes that mediate this contextual difference may have been too subtle to be revealed with BOLD fMRI or may require more statistical power, both within subjects and in terms of group sample size. This null finding will have to be rejected or replicated in future behavioral and neuroimaging studies.

In previous studies, the medial aspect of the prefrontal cortex has been associated with theory of mind (Happé et al., 1996; Adolphs, 2001), socially-complex, person-perceiving mentalizing tasks (Gallagher and Frith, 2003; Frith and Frith, 2006, 2012; Frith, 2007), and inferring information about the emotional state of another person (Ochsner et al., 2004; Mitchell et al., 2005; Saxe and Powell, 2006; Krämer et al., 2010). Mitchell et al. (2005) have proposed a simulation theory in which activity in these regions may be associated with the prediction of others' states based on a subject's own experience (see also Lombardo et al., 2010). Indeed, recent research has fine-tuned this account to propose a spatial mentalizing gradient in this brain area, associating the capacity to make judgments about self and others with a distributed gradient along the ventral to dorsal parts of the medial prefrontal cortex (Denny et al., 2012). In addition, Coricelli and Nagel (2009) showed that the medial prefrontal cortex is recruited by individuals using high-level reasoning (and expecting similar forms of reason from others) in a competitive context, thus supporting our suggestion that truth telling is associated with brain-areas related to complex and socially-mediated high-level cognition, and not always suitable as a control condition from which cognitively-taxing deception conditions can be disaggregated. Thus, we suggest that in conditions of socially-stressful truth telling, medial prefrontal activity may encode effects of our own choices and actions onto the thought and behavior of others. If Theory of Mind describes the capacity to attribute mentality to another, empathy is the ability to see a similarity between one's own feelings and those of another (Decety and Jackson, 2004). This distinction is important for our study, where the design (participants assess others' poor performance, in the belief that they, too, will be the subjects of others' assessments) is sensitive to empathizing, in addition to Theory of Mind. Activation found in the anterior insula of our sample may also indicate feelings of empathy, and a sense of fellow-feeling from participants who recognize that telling an unpleasant social truth may have effects on another person, (Bernhardt and Singer, 2012). In situations involving social truth telling, the speaker must manage, not only information about the question and content, but also the potential costs of revealing truths about another person. It has also been suggested that the medial prefrontal area is involved in planning for the future. Frith and Frith (2006) suggest that activity in this area may be involved in predicting how others will think and feel, and in planning for the outcome of their experience of particular states. Our results support the interpretation that being asked to tell the truth requires an individual to take another person's perspective and to empathize in order to predict how others will think and feel.

## Limitations and future directions

This study engages a new research area: socially stressful truth telling. As such, it contains several important limitations, each of which could be overcome in future experiments. First, while our experimental design is counterbalanced for perceived gender differences, there is a potential gender imbalance among our participant group (27 participants, 21 females) that may have affected the ingroup/outgroup dynamics. Our sample population was chosen from local choir singers, who are statistically more likely to be female (although choir singing remains a rare example of a competitive group activity in which make and female participants compete together). Future studies could include an equal number of male and female participates in the study. Second, because this study worked within a novel context of “socially stressful truth,” we did not have statistically–validated question sets from which to choose. To develop our question set we ran a pilot study that consisted of a survey (completed by participants with similar demographic profiles to the final study participants) on the social-stress level of 40 questions, from which we chose the 20 most stressful questions. Future studies could benefit from our question bank and from more validated research into the range of questions that subjects in a particular group might identify as socially stressful. Third, we did not test the level of honesty among participants while they were undergoing scanning. We were interested in activation caused by *being asked* to tell a socially stressful truth, not in activation caused by *telling* a socially stressful truth. Nonetheless, follow-up studies—particularly those that wish to focus on connections between activations caused by lying and activations caused by truth telling—would likely incorporate new sets of questions that might be measured against a truth/deception scale and/or incorporate a set of pre and post interview questionnaires that would help to measure how truthful (or deceptive) the participant perceived themselves to be during the experimental scanning.

## Conclusion

The results of this study indicate that “truth” is not a simple or singular variable. We show that participants have slower reaction times and differential activation of the medial prefrontal cortex, anterior insula, and precuneus when asked to tell a social truth. Future research is needed to characterize the neuronal mechanisms that mediate various truth telling conditions, which could range from simple truth to social truth and beyond. Future studies might also take account of the potential neurobiological overlap between truth and lie conditions, which may pose a confounding effect on the isolation of either “truth” or “deception.” Our data suggests that being asked to tell a social truth may be mediated by context; and that certain kinds of truths are more consequential than other truths.

## Funding

This work was supported by the European Neuroscience and Society Network (ENSN); the Danish Agency for Science, Technology and Innovation's University Investment Grant to MIND*lab*, Aarhus University; the University of Illinois Research Board, and the Center for Advanced Study and the Kinesiology and Community Health Department at the University of Illinois, Urbana-Champaign. This work was also supported by the Wellcome Trust [103817/Z/14/Z].

### Conflict of interest statement

The authors declare that the research was conducted in the absence of any commercial or financial relationships that could be construed as a potential conflict of interest.
